# In vivo modification of Abeta plaque toxicity as a novel neuroprotective lithium-mediated therapy for Alzheimer’s disease pathology

**DOI:** 10.1186/2051-5960-1-73

**Published:** 2013-11-12

**Authors:** Laura Trujillo-Estrada, Sebastian Jimenez, Vanessa De Castro, Manuel Torres, David Baglietto-Vargas, Ines Moreno-Gonzalez, Victoria Navarro, Raquel Sanchez-Varo, Elisabeth Sanchez-Mejias, Jose Carlos Davila, Marisa Vizuete, Antonia Gutierrez, Javier Vitorica

**Affiliations:** 1 Departamento de Biologia Celular, Genetica y Fisiologia, Facultad de Ciencias, Universidad de Malaga, 29071 Malaga, Spain; 2 Departamento de Bioquímica y Biologia Molecular, Facultad de Farmacia, Universidad de Sevilla, 41012 Sevilla, Spain; 3 Centro de Investigación Biomédica en Red sobre Enfermedades Neurodegenerativas (CIBERNED), Madrid, Spain; 4 Instituto de Biomedicina de Sevilla (IBiS)-Hospital Universitario Virgen del Rocío/CSIC, Universidad de Sevilla, Sevilla, Spain; 5 Present addresses: Laboratory of Molecular Cell Biomedicine, University of Balearic Islands, Palma, de Mallorca, Spain; 6 Institute for Memory Impairments and Neurological Disorders, Department of Neurobiology and Behavior, University of California, Irvine, CA, USA; 7 Mitchell Center for Alzheimer’s Disease and Related Brain Disorders, Department of Neurology, University of Texas Medical School at Houston, Houston, Texas, USA

**Keywords:** Alzheimer, Lithium treatment, Transgenic mice, Neuronal degeneration, Axonal dystrophies, Abeta plaques, Toxicity

## Abstract

**Background:**

Alzheimer’s disease (AD) is characterized by the abnormal accumulation of extracellular beta-amyloid (Abeta) plaques, intracellular hyperphosphorylated tau, progressive synaptic alterations, axonal dystrophies, neuronal loss and the deterioration of cognitive capabilities of patients. However, no effective disease-modifying treatment has been yet developed. In this work we have evaluated whether chronic lithium treatment could ameliorate the neuropathology evolution of our well characterized PS1M146LxAPPSwe-London mice model.

**Results:**

Though beneficial effects of lithium have been previously described in different AD models, here we report a novel in vivo action of this compound that efficiently ameliorated AD-like pathology progression and rescued memory impairments by reducing the toxicity of Abeta plaques. Transgenic PS1M146LxAPPSwe-London mice, treated before the pathology onset, developed smaller plaques characterized by higher Abeta compaction, reduced oligomeric-positive halo and therefore with attenuated capacity to induce neuronal damage. Importantly, neuronal loss in hippocampus and entorhinal cortex was fully prevented. Our data also demonstrated that the axonal dystrophic area associated with lithium-modified plaques was highly reduced. Moreover, a significant lower accumulation of phospho-tau, LC3-II and ubiquitinated proteins was detected in treated mice. Our study highlights that this switch of plaque quality by lithium could be mediated by astrocyte activation and the release of heat shock proteins, which concentrate in the core of the plaques.

**Conclusions:**

Our data demonstrate that the pharmacological in vivo modulation of the extracellular Abeta plaque compaction/toxicity is indeed possible and, in addition, might constitute a novel promising and innovative approach to develop a disease-modifying therapeutic intervention against AD.

## Background

In Alzheimer’s disease (AD), the abnormal accumulation of extracellular beta-amyloid (Abeta) plaques and intracellular hyperphosphorylated tau induces progressive synaptic alterations, axonal dystrophies, neuronal loss and the deterioration of cognitive capabilities of patients [1,2]. In spite to the relatively large information about the AD pathology, no effective disease-modifying treatment has been yet developed. Within the different compounds tested, lithium, a primary drug to treat bipolar disorder, has also been suggested as a potential treatment against AD [3-6]. In fact, clinical studies indicated that lithium could be preventive in patients with MCI, whereas no beneficial effects were observed in mild to moderate AD [4]. In addition, epidemiological studies also reported a reducing risk of AD in patients with bipolar disorders treated with Li [5]. Thus, lithium may indeed constitute a useful preventive treatment for individuals at high risk of AD and/or preclinical stages of the disease.

The neuroprotective mechanisms of lithium are not completely understood. In AD models, lithium could reduce the AD pathology inhibiting (directly and/or indirectly) the activity of the tau kinase GSK-3beta. Whereas this inhibition would preclude tau phosphorylation [7,8], dystrophy formation and neuronal degeneration [9,10], the therapeutic benefits of this treatment have been questioned [11]. On the other hand, lithium also mediated the inhibition of inositol monophosphatase and the induction of mTOR-independent autophagic process [12,13]. This induction may be important in the prevention or attenuation of neurodegeneration associated with aggregated proteins. Moreover, since the accumulation of autophagic vesicles could also be implicated in the formation of axonal dystrophies in AD models [14-17], lithium should alleviate the progression of these pathological features. However, while several studies have shown beneficial effects in lowering Abeta load [18-20], others reported no effect or even increased Abeta production [11,21,22].

The origin of these controversies is currently unknown. Among other factors, the different AD models, the different protocols of lithium administration or dosage and, perhaps more relevant, the partial neuropathology displayed for most of the AD models, could explain the discrepancies between the different effects of lithium treatment in transgenic models.

In this work we have evaluated the effect of chronic oral lithium treatment using the bigenic PS1M146LxAPPSwe-London mice. This model displayed early degeneration of O-LM and HIPP interneurons (SOM/NPY-positive), in hippocampus and entorhinal cortex [23,24]. These GABAergic cells are implicated in memory/learning processes and degenerate in AD patients [25,26]. Our data demonstrated that chronic (from 3- to 9-month-old) oral lithium administration, initiated before the onset of Abeta deposits, efficiently prevented most of the early neuropathological manifestations of our PS1xAPP model. Lithium prevented the neuronal loss (at both hippocampus and entorhinal cortex), reduced the tau phosphorylation and the formation of axonal dystrophies and, in consequence, ameliorated behavioral/memory deficits observed at this age. These effects were mediated by increasing the compaction of Abeta plaques and lowering their toxic oligomeric halo. This modification on Abeta deposits toxicity is a novel disease-modifying effect of lithium, acting through the astrocytes and the release of heat shock proteins (Hsps).

## Methods

### Transgenic mice and lithium treatment

Generation of PS1M146LxAPP751Swe-London (PS1xAPP) mice has been reported previously [27]. Heterozygous PS1xAPP double transgenic mice (C57BL:6 background) were generated by crossing homozygous PS1 transgenic mice with heterozygous Thy1-APP751SL mice. Only male mice were used in this work.

**Figure 1:**
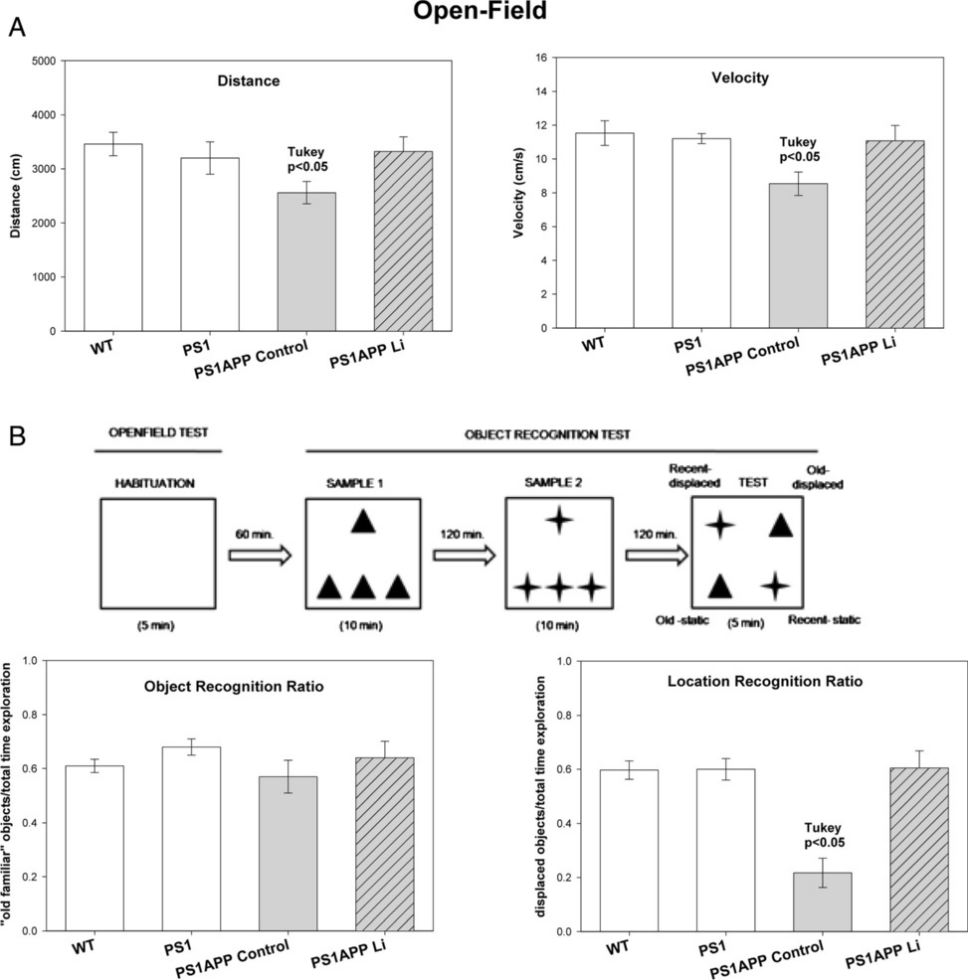
**Lithium treatment improved the behavioral/memory deficiencies of PS1xAPP mice. (A)** Open-field test. One-way ANOVA revealed significant differences between groups in both distance (F(3,24) = 6.33; p = 0.006) and velocity F(3,24) = 8.02, p = 0.002). Post-hoc analysis showed significant differences in PS1xAPP control compared with WT/PS1 and PS1xAPP lithium (p < 0.05). **(B)** Behavioral protocol and memory measures. Sixty min after habituation to the open-field, animals received three samples trials with 120 min inter-trial as depicted. Each symbol (triangle and star) represents one type of object. One-way ANOVA F(3,24) = 21.15 p = 0.0001) revealed significant differences between groups in place memory index. Post-hoc analysis showed significant difference in PS1xAPP control compared with WT/PS1 controls and PS1xAPP lithium.

**Figure 2:**
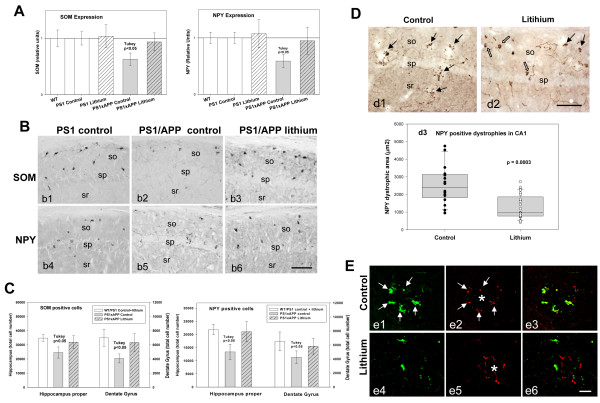
**Lithium treatment avoided neuronal loss in the hippocampus of PS1xAPP mice. (A)** The expression of SOM and NPY (n= 20 mice per group) was significantly lower in PS1xAPP control group (ANOVA F(4,96) = 49.63, p = 0.00001. Tukey p < 0.05 or F(4,96) = 27.24, p = 0.0001, Tukey p < 0.05 for SOM and NPY respectively). WT, PS1 (control or lithium) and PS1xAPP lithium displayed no differences. **B)** SOM (b1-b3) or NPY (b4-b6) immunolabeled sections through CA1 subfield of PS1 control or PS1xAPP control and lithium groups. **C)** Total SOM or NPY cell number in hippocampus proper and dentate gyrus was assessed by stereology (n = 5 per genotype and treatment). WT and PS1 groups displayed no differences and data were pooled. Only control PS1xAPP displayed a significant reduction in SOM (ANOVA F(2,22) = 14.57, p = 0.00002 or F(2.22) = 11.84, p = 0.0008 for hippocampus proper and dentate gyrus, respectively) and NPY (ANOVA F(2,22) = 17.02, p = 0.0001 or F(2,22) = 6.87, p = 0.008 for hippocampus proper and dentate gyrus, respectively) cell number. **D)** NPY-positive dystrophies (black arrows) in CA1 subfield of PS1xAPP control (d1) and lithium mice (d2) (open arrows: NPY cell bodies). (d3) Quantitative analysis (n = 4 mice per group; 6 sections per animal) demonstrated the existence of a significant reduction of NPY-positive dystrophic area (μm2) in PS1xAPP lithium. **E)** Co-localization of SOM (green) and AT8 (red) in dystrophies assessed using confocal microscopy. In PS1xAPP control mice (e1 to e3), SOM positive dystrophies (arrows), surrounding Abeta plaque (asterisk), were positive for AT8. Lithium produced a reduction in both SOM (e4) and AT8 (e5) positive dystrophies and in the co-localization between SOM and AT8 in dystrophies (e6). Scale bars: b1-b6 100 μm; d1 and d2 100 μm; e1-e6 20 μm.

As controls, we used age-matched non-transgenic (C57BL:6) male mice (WT) or hemizygous PS1M146L littermates (PS1). The PS1 mice displayed no apparent differences with WT mice, at the age used in this work [24] (see also Figures 1 and 2A). Only PS1xAPP mice model accumulated Abeta plaques. Thus, to specifically assess the potential therapeutic effect of lithium on the Abeta pathology, we reasoned that PS1 mice would be a better control than WT mice. An additional lithium treated PS1 group was also used as control for lithium treatment.

For lithium treatment, PS1 and PS1xAPP mice (3 month old at the beginning of treatment) were randomly divided into two groups (n = 25 each). Mice were fed, ad libitum, with standard mice diet (2014 Teklad Global 14% Protein Rodent Maintenance Diet, Harlan, Spain) or standard mice diet supplemented with lithium carbonate (1.2 g/kg 2014 diet, Harlan, Spain). The lithium group received an additional drinking bottle containing NaCl (0.7%). The treatment was continued for 6 months. The control and treated mice were weighted weekly and no significant weight loss was detected (29.20 ± 1.09 g vs 27.5 ± 2.2 g for control and Li-treated PS1xAPP mice, respectively). For the plasmatic Li content, mice were bled (100 μl) from the ocular artery. The plasmatic lithium levels were stable during the treatment and also were within the therapeutic range: 0.44 ± 0.07 mEquiv/L (n = 10) after 71 days of treatment or 0.38 ± 0.05 mEquiv/L (n = 10) at the end of treatment. This treatment was well tolerated with a low mortality during this period. (PS1 control 0%; PS1 lithium, 0%; PS1xAPP control, 20%; PS1xAPP lithium, 7.4%).

After behavioral tests, control and lithium-treated mice were anesthetized with sodium pentobarbital (60 mg/kg), and transcardially perfused with 0.1 M phosphate buffered saline (PBS). Then, mice brain was quickly removed and one hemibrain dissected (cortex and hippocampus), frozen and stored at −80°C while the other hemibrain was fixed by immersion with 4% paraformaldehyde, 75 mM lysine, 10 mM sodium metaperiodate in 0.1 M phosphate buffer (PB), pH 7.4 for 5 days at 4°C. Fixed hemibrains were cryoprotected in 30% sucrose, sectioned at 40 μm thickness in the coronal plane on a freezing microtome and serially collected in wells containing cold PBS and 0.02% sodium azide.

All animal experiments were performed in accordance with the animal research regulations (RD53/2013 and 2010/63/UE) from Spain and European Union, and with the approval of the Committees of Animal Research from the University of Seville (Spain) and the University of Malaga (Spain).

### Behavioral studies

All experiments were conducted on age-matched male WT (n = 10), PS1 (n = 10), PS1xAPP control (n = 9) and PS1xAPP lithium-treated (n = 9) mice. Mice were tested at 9 month of age, two days previous to sacrifice. All testing were performed in the light period of the light/dark cycle and the experimenter was blind to the genotypes and treatment of mice. Animals were adapted to the experimental conditions for 6 days before behavioral testing. All mice were moved to the testing room in their home-cage and kept in the room during 1 hour to habituate to the new location; then, they were handled gently each day briefly in order to minimize non-specific stress. The behavioral experiment protocol was conducted as shown in Figure 1.

#### Open-field test

Besides the use of the open-field to habituate animals to the cage before the object recognition test, we examined motor function by means of spontaneous locomotor activity [28]. In this test, mice were placed in the centre of a square-shape arena (45 cm × 45 cm) and were allowed to explore the arena for 5 minutes. The arena was thoroughly cleaned with 70% ethanol solution after each trial. The locomotor activity was measured by an automated monitoring system (Ethovision, Noldus, The Netherlands). Distance travelled and velocity was quantified.

#### Object recognition test

The object recognition test is based in the natural tendency of rodents to explore objects (spontaneous exploratory behavior). In the present study, we used a modified protocol [29] based in the preference for the “old familiar object” over the “recent familiar object” and the preference for a novel location. Sixty minutes after habituation to the open-field, animals were first exposed to four identical objects arranged in a triangle shaped configuration and allowed to explore them for 10 minutes (Sample 1). After a delay of 2 hours, the mice received a second sample trial identical to the first, except that a novel set of four identical objects were present (Sample 2). The test trial started after 2 hours interval and lasted for 5 minutes. In the test trial, two objects from both samples 1 and 2 (“old familiar” and “recent familiar” objects, respectively) were arranged in a quadratic shape configuration, so one old object and one recent object were present in a familiar position while the other two were displaced to a new position (see Figure 1B for details). The type of object used as “old” and “recent” was counterbalanced across mice. All objects were made of plastic to prevent material preference and for an easier cleaning to prevent odor cues. The two sets of objects were different in size, form and color. The arena and objects were thoroughly cleaned with 70% ethanol solution after each trial. The time spent by the mice exploring each object was analyzed observationally. Indeed, the basic measure was the time spent by the mice exploring objects during the sample phases and during the test trial. Additionally, two discrimination indexes were calculated for the test trial: an object recognition ratio (total time exploring “old familiar” objects/total time exploration) and a location recognition ratio (total time exploring displaced objects/total time exploration). The time was recorded only when the mice touched the object with its nose or forepaws. Turning around, walking over the object, rearing above the object or resting close to the object was not deemed to be exploration. Moreover, locomotor activity was also measured with the software Ethovision XT 7.0 (Noldus, The Netherlands).

### RNA and total protein extraction

Total RNA from mice hippocampi was extracted using Tripure Isolation Reagent (Roche) as described previously [23,24,30,31]. After isolation, RNA integrity was assessed by agarose gel electrophoresis. The yield of total RNA was determined by measuring the absorbance (260:280 nm) of isopropanol-precipitated aliquots of the samples. The recovery of RNA was comparable in all studied groups (1.2-1.5 μg/ mg of tissue). The protein pellets, obtained using the Tripure Isolation Reagent and isopropanol-mediated precipitation, were resuspended in 4% SDS and 8 M urea in 40 mM Tris–HCl, pH 7.4 and rotated overnight at room temperature to get complete protein solubilization.

### Retrotranscription and real-time RT-PCR

Retrotranscription (RT) was performed using random hexamers, 4 μg of total RNA as template and High-Capacity cDNA Archive Kit (Applied Biosystems) following the manufacturer recommendations [24,30]. For real time RT-PCR, commercial TaqmanTM probes (Applied Biosystems) were used for amplification. PCR reactions were carried out using either ABI Prism 7000 or 7900HT sequence detector systems (Applied Biosystems). A standard curve was first constructed for every assay, using increasing amounts of cDNA. In all cases, the slope of the curves indicated optimal PCR conditions (slope 3.2-3.4). The cDNA levels of the different mice were determined using GAPDH as housekeeper. Therefore, GAPDH amplification was done in parallel with the gene to be analyzed, and this dada used to normalize target gene results.

Independently of the analyzed gene, results were always expressed using the comparative Ct method, following the Bulletin number 2 from Applied Biosystems. As a control condition, we selected 9 month-old WT mice with control diet. In consequence, the expression of all tested genes, for all ages and mice types, was referenced to the expression levels observed in this group.

### Antibodies

For this study the following primary antibodies were used: anti-Neuropeptide Y (NPY) rabbit polyclonal (1:5000, Sigma); anti-Somatostatin (SOM) goat polyclonal (1:1000, Santa Cruz Biotechnology); anti-Abeta (clone 6E10) mouse monoclonal (1:5000, Signet); anti-oligomeric amyloid-beta OC rabbit polyclonal (1:5000, Millipore); anti-Abeta42 rabbit polyclonal (1:5000, Abcam); anti-phospho-tau pSer202/Thr205 mouse monoclonal (clone AT8) (1:250, Pierce); anti-microtubule-associated protein 1 light chain 3 (LC3) rabbit polyclonal (1:500, Cell Signaling); anti-GFAP rabbit polyclonal (1:10000, Dako); anti-ubiquitin rabbit polyclonal (1:5000, Dako); anti-Hsp70 rabbit polyclonal (1:5000, Neomarkers) anti-Hsp60 mouse monoclonal (1:1000, Santa Cruz, Biotechnology), anti-Hsp27 rabbit polyclonal (1:1000, Sigma).

### Western blot

Western blots were performed as described [32]. Briefly, 5–20 μg of proteins from the different samples were loaded on 16%-SDS-tris-tricine-PAGE or 12%-SDS-tris-glycine-PAGE and transferred to nitrocellulose (Hybond-C Extra; Amersham). After blocking, using 5% non-fat milk, membranes were incubated overnight, at 4°C, with the appropriate antibody Membranes were then incubated with the corresponding horseradish-peroxidase-conjugated secondary antibody (Dako, Denmark) at a dilution of 1:8000. Each blot was developed using the ECL-plus detection method (Amersham) and quantified using ImageQuant Las 4000 mini gold (GE Healthcare Bio-Sicences). For normalization purposes, proteins were first estimated by Lowry and protein loading corrected by beta-actin. In each experiment, the intensity of bands from PS1 control fed were averaged and considered as 1 relative unit. Data were always normalized by the specific signal observed in PS1 control group.

### Immunohistochemistry

Serial sections from control and lithium-treated transgenic mice (n = 6 per group) were processed in parallel for immunostaining using the same batches of solutions to minimize variability in the immunohistochemical labeling conditions. Free-floating sections were first treated with 3% H2O2/10% methanol in PBS, pH 7.4 for 20 min to inhibit endogenous peroxidases, and with avidin-biotin Blocking Kit (Vector Labs, Burlingame, CA, USA) for 30 min to block endogenous avidin, biotin and biotin binding proteins. Sections were immunoreacted with the primary antibody over 24 or 48 h at room temperature. The tissue bound primary antibody was then detected by incubating for 1 h with the corresponding biotinylated secondary antibody (1:500 dilution, Vector Laboratories), and then followed by incubating for 90 min with streptavidin-conjugated horseradish peroxidase (Sigma-Aldrich) diluted 1:2000. The peroxidase reaction was visualized with 0.05% 3-3-diaminobenzidine tetrahydrochloride (DAB, Sigma-Aldrich) and 0.01% hydrogen peroxide in PBS. Except for Abeta42 immunolabeling, the chromogen solution contained 0.03% nickel ammonium sulphate for a blue reaction product. For double NPY-6E10 immunohistochemical labeling sections were first incubated with anti-NPY as described above. After the DAB-nickel reaction (dark blue end product), sections were the incubated with anti-6E10 antibody. The second immunoperoxidase reaction was developed with DAB only (brown reaction end product). After DAB, sections immunolabeled for Ubiquitin or LC3 antibodies were incubated 3 min in a solution of 20% of Congo red. Sections were then mounted on gelatin-coated slides, air dried, dehydrated in graded ethanol, cleared in xylene and coverslipped with DPX (BDH) mounting medium. Specificity of the immune reactions was controlled by omitting the primary antisera.

For double or triple immunofluorescence labelings, sections were first sequentially incubated with the indicated primaries antibodies followed by the corresponding Alexa 488/568//405 secondary antibodies (1:1000; Invitrogen). GFAP- and OC-immunolabeled sections were stained with 0.02% thioflavin-S in 50° ethanol for 5 min. Sections processed for immunofluorescence were mounted onto gelatin-coated slides, coverslipped with 0.01M PBS containing 50% glycerin and then examined under a confocal laser microscope (Leica SP5 II).

### Stereological analysis

Immunopositive cells for SOM or NPY belonging to control and lithium-treated animal groups (n = 5 per group) were stereologically quantified in hippocampus proper and dentate gyrus of hippocampus according to the optical fractionator method. Briefly, the quantitative analyses were performed using an Olympus BX61 microscope interfaced with a computer and an Olympus DP71 digital camera, and the NewCAST (Computer Assisted Stereological Toolbox) software package (Olympus, Denmark). Cell counting was done through the rostrocaudal extent of the hippocampus (between −0.94 mm anterior and 3.64 mm posterior to Bregman coordinates). Neurons were quantified in every seventh section (with a distance of 280 μm), and an average of 6–7 sections was measured in each animal. CA and dentate gyrus boundaries were defined according a standard mouse stereotaxic brain atlas using a 4x objective and the number of neurons was counted using a 100×/1.35 objective. We used a counting frame of 1874.2 μm2 with step lengths of 78.93 × 78.93 μm. The total cell number was estimated using the optical fractionators formula, N = 1/ssf × 1/asf × 1/hsf × ∑ Q ‒, where *ssf* represents the section sampling fraction, *asf* is the area sampling fraction, which is calculated by dividing the area sampled with the total area of the layer, *hsf* stands for the height sampling fraction, which is calculated by dividing the height sampled (10 μm in this study) with the section thickness, and ∑Q- is the total count of somatic profiles counted for the entire area. The precision of the individual estimations is expressed by the coefficient of error (CE) using the following formula: CE  = 1/Q × (3A ‒ 4B + C/12)1/2, where A = ∑Qi2, B = ∑Qi × Qi + 1, C = ∑Qi × Qi + 2. The CEs ranged between 0.07 and 0.1. An investigator who was blind to the experimental conditions performed neuronal profile counting.

### Plaque size

To determine the size of the plaques, anti-Abeta42 immunostained sections from control and lithium-treated mice (n = 6 per group) were analyzed using the nucleator method with isotropic probes by the NewCAST software package from Olympus stereological system. CA1 subfield was analyzed using a counting frame of 7155.3 μm2. For individual plaque measurement a 40x objective was used. Number of plaques/mm2 falling into surface categories (ranging from <200 μm2 to >2000 μm2) was calculated. Each analysis was done by a single examiner blinded to sample identities.

### NPY dystrophic neurites loading

NPY immunostained sections from control and lithium-treated animals were observed under a Nikon Eclipse 50i microscope using a 10x objective and CA1 images were acquired with a Nikon DS-5M high-resolution digital camera. The camera settings were adjusted at the start of the experiment and maintained for uniformity. Digital images (5 sections/mouse) from control and treated mice (n = 6 per group) were analyzed using Visilog 6.3 analysis program (Noesis, France). The area occupied by the NPY-positive dystrophic neurites was identified by level threshold which was maintained throughout the experiment for uniformity. The CA1 area in each image was manually outlined and the positive somata were removed by manual editing. The area occupied by NPY dystrophies was estimated and defined as (sum dystrophies area measured/sum CA1 area analyzed) × 100. The mean and standard deviation (SD) of the dystrophies area were determined using all the available data. Quantitative comparisons were carried out on sections processed simultaneously using same batches of solutions.

### NPY dystrophies associated to plaques

The area of NPY dystrophic neurites intimately associated to plaques of different sizes (<200 μm2, 200–500 μm2, 500–2000 μm2 and >2000 μm2) was measured in double 6E10/NPY immunostained CA1 sections from control and lithium-treated animals. Images were photographed using a 20x objective with a Nikon Eclipse 50i microscope coupled to a Nikon DS-5M high-resolution digital camera. Digital images (5 sections/mouse) from control and lithium-treated animals (n = 3 per group) were analyzed using Visilog 6.3 analysis program (Noesis, Frace) to determine the NPY dystrophies area associated to each plaque size group.

### Plaque compaction analysis

Abeta42 immunostained hippocamapal sections from control and lithium-treated animals were observed under a Nikon Eclipse 50i microscope and CA1 plaques were photographed using a 10x objective. Images were acquired with a Nikon DS-5M high resolution digital camera. The camera settings were adjusted at the start of the experiment and maintained for uniformity. Digital images (5 sections/mouse, n = 5 per group) were analyzed using Visilog 6.3 analysis program (Noesis, France). Abeta42 staining density was identified by bright-level threshold, the level of which was maintained throughout the experiment for uniformity. The gray-scale image was converted to a binary image for estimating the optical density which was defined as pixel units and related with the plaque size (μm2 which area was measured using the same program). Quantitative comparisons were performed on sections processed at the same time.

### Oligomeric plaque halo

To analyze the oligomeric Abeta halo located at the periphery of the plaques, 40 μm floating sections were first stained with Thioflavin-S and then followed by an antibody specific to oligomers of Abeta (OC antibody; 1:5000) visualized with anti-rabbit Alexa568-conjugated secondary antibody (Invitrogen A10042; 1:1000). Images of 1,024 × 1,024 pixels were acquired by using a Leica SP5 II confocal laser microscope. A total of 15 plaques per animal were randomly photographed in CA1 subfield of control and treated animals (n = 3 per group). Laser settings were adjusted at the start of the experiment and maintained for uniformity. Images were analyzed using LAS AF Lite program (Leica). Plaque area was determined for Thioflavin-S staining (plaque core in green color) and OC immunostaining (oligomeric Abeta in red color) and the difference between the OC area and the core area was considered as the oligomeric halo surrounding plaques.

### Statistical analysis

Normality of data was first assessed by using Kolmogorov-Smirnov test. Normally distributed data were expressed and represented as mean ± SD. Non-normal distributed data were represented using box-plot. For normally distributed data, means were compared using ANOVA followed by Tukey test (more than two groups) or t-test (for two group comparisons). Non-normal data were compared by Wilcoxon (for two groups) or Kruskal-Wallis tests (more than two groups). The significance was set at 95% of confidence. Fit and comparison of linear regression was done using multiple regression analysis followed by conditional sum of squares. In all cases Statgraphics plus 3.1 was used.

## Results

### Lithium treatment rescued behavioral/memory deficits

First, we tested whether lithium administration was able to improve the behavioral/memory deficiencies observed in the 9 month-old control PS1xAPP transgenic model. From the different tasks performed (i.e. Morris water maze), only open-field and novel object recognition showed statistical differences between PS1xAPP and WT or PS1 control mice. No differences between WT and PS1 mice were observed.

In the open-field test, control PS1xAPP mice displayed significant lower activity than either WT or PS1 mice (Figure 1A). There was a significant reduction in both total distance and velocity. No significant differences were observed in time in the periphery or center of the field, or in the immobility periods (data not shown). Also, no differences between PS1xAPP and control groups were observed in fecal boli depositions (not shown). Thus, the PS1xAPP mice were hypoactive as reported in other AD models, such as the 3xTg-AD [28]. As we shown, this mild form of apathy in PS1xAPP mice was totally relieved after lithium treatment (Figure 1A).

We next evaluated the episodic-like memory using novel object recognition tests. These tests are based on the preference for the “old familiar object” over the “recent familiar” object and the preference for a novel location [29]. Although no differences between groups were observed in the object recognition ratio (Figure 1B), control PS1xAPP mice displayed a significant cognitive deficit in object location memory (which is hippocampus-dependent), compared with PS1/WT mice. Remarkably, full recovery of this spatial memory impairment was observed in lithium-treated PS1xAPP mice, which displayed no differences with the control groups (Figure 1B). Therefore, these data indicated that early oral lithium administration prevents the spatial memory deterioration in PS1xAPP mice.

### Lithium administration prevented neuronal loss

As we have reported previously, early Abeta deposition in this PS1xAPP model is paralleled by a selective and significant decrease in the number of SOM and NPY positive GABAergic neurons in the hippocampal formation and the entorhinal cortex [23,24], (see also Figure 2A, B and C). Furthermore, SOM/NPY positive cells displayed a prominent axonal pathology (Figure 2D), with multiple dystrophies (positive also for phospho-tau) that surrounded Abeta plaques (Figure 2E). The early and extensive degenerative pathology of the GABAergic cells, including neuronal death, could be used as a surrogated marker to evaluate the neuroprotective effect of lithium at the initial stages of the disease.

We thus analyzed, at the hippocampal formation, the SOM and NPY expression by qPCR; the number of SOM or NPY immunopositive somata, by stereological quantification, and their axonal dystrophy loading. Lithium produced an effective prevention of the neurodegenerative process exhibited by this neuronal population in PS1xAPP mice (Figure 2). In fact, the mRNA expression for both neuropeptides, SOM and NPY, was virtually identical in lithium-treated PS1xAPP mice, as compared with control groups (Figure 2A). Furthermore, stereological determination of the SOM or NPY cell number (Figure 2B and C) further demonstrated the protective effect of lithium on these neurons in hippocampus proper (O-LM cells) and dentate gyrus (HIPP cells). PS1 and WT mice displayed not differences and lithium treatment of PS1 mice did not alter the expression of either SOM or NPY neuropeptides (see Figure 2A) or the number of SOM or NPY positive cells (data in Figure 2C were pooled from WT, PS1 control and lithium groups). For subsequent experiments, only PS1 control and PS1 lithium mice were included.

The same samples were used for the stereological quantification of SOM or NPY cell number at the entorhinal cortex, another early affected brain region. As previously reported [23], double transgenic mice displayed in this cortical region a significant decrease in the number of both SOM (4,207 ± 1,119 cell/mm3, n = 4, vs 8,596 ± 803 cell/mm3, n = 6, for control PS1xAPP and PS1 mice, respectively, p < 0.05; 52.2 ± 13.1% of reduction) and NPY (1,770 ± 227 cell/mm3, n = 4, vs 3,062 ± 363 cell/mm3, n = 6, for control PS1xAPP and PS1 mice, respectively, p < 0.05; 58.4 ± 7.1% of reduction) immunopositive GABAergic cells. However, after lithium treatment, the number of both neuronal populations did not differ between PS1/APP and PS1 groups (7,087 ± 1,097 cell/mm3 or 3,003 ± 423 cell/mm3, n = 4, for SOM and NPY, respectively; 17.6 ± 12.8% or 1.9 ± 13.8% of reduction).

Thus, early lithium intervention was highly effective preventing the SOM/NPY neuronal loss in both the hippocampal formation and the entorhinal cortex of PS1xAPP model.

Another prominent pathological feature of this neuronal population in our PS1xAPP mice is the extensive development of axonal dystrophies associated to Abeta plaques [15] (Figure 2D and E). Importantly, lithium treatment produced, in parallel with the prevention of the SOM/NPY cell loss, a prominent reduction of the axonal dystrophy pathology in these cells. In fact, lithium treated PS1xAPP mice displayed an obvious and highly significant decrease (−60.2%) in the NPY positive dystrophic area (Figure 2D, d1, d2 and d3). Also, there was a decrease in the AT8- and SOM-positive dystrophies (Figure 2E). Therefore, lithium avoided SOM/NPY neurodegeneration and improved the cell integrity, reducing the axonal degeneration of this highly vulnerable neuronal population.

### Lithium treatment ameliorated axonal/synaptic pathology by reducing abnormal intracellular protein accumulation

**Figure 3:**
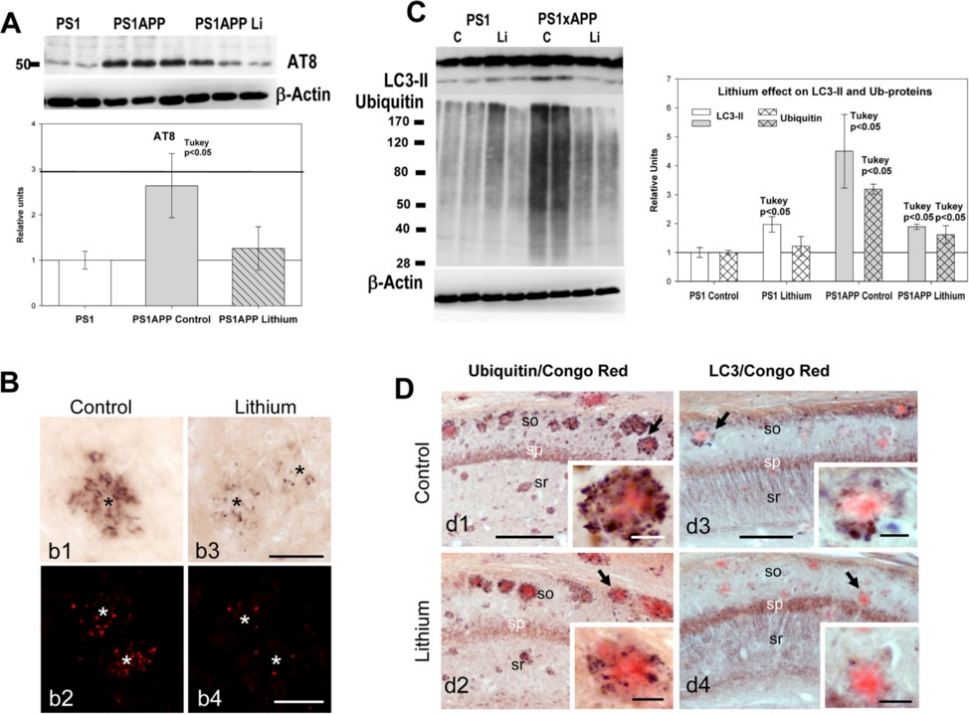
**Lithium treatment reduced the dystrophic pathology associated to Abeta plaques. A)** Representative western blot (n = 7 mice per group) and quantitative analysis of phosphorylated tau, determined using AT8 clone, of total proteins from PS1 control, PS1xAPP control and PS1xAPP lithium-treated mice. For quantification, the AT8 levels were referred to PS1 control group. The AT8 levels were significantly increased in PS1xAPP control mice (ANOVA F(2,18) = 22.66, p = 0.0001; Tukey p < 0.05) whereas PS1 control and PS1xAPP lithium displayed no differences. **B)** Representative AT8 positive dystrophies surrounding Abeta plaques from control (b1 and b2) or lithium treated (b3 and b4) PS1xAPP mice. **C)** Representative western blots and quantitative analysis of steady-state levels of LC3-II and ubiquitinated proteins in PS1 and PS1xAPP control and treated mice (n = 4 per genotype and treatment). A clear and significant accumulation of both LC3-II (ANOVA F(3,12) = 32.52, p = 0.001) and ubiquitinated proteins (ANOVA (F(3,12) = 63.67, p = 0.00001) was observed in PS1xAPP control mice. Lithium treatment reversed this pathology. The post-hoc analysis, using Tukey test, was indicated in the figure. **D)** Ubiquitin (d1 and d2) and LC3 (d3 and d4) immunolabeled hippocampal sections (counterstained with Congo red for Abeta plaques) of control and lithium treated PS1xAPP mice corroborated the accumulation of both markers, associated with dystrophic neurites surrounding amyloid plaques (d1 and d3) and the patent lithium reduction (d2 and d4) in the presence of ubiquitin and LC3 positive dystrophies. Inserts in d1-d4 showed higher magnification details of the dystrophies surrounding Abeta plaques. so, stratum oriens; sp, stratum pyramidale; sr, stratum radiatum. Scale bars: b1, b3 50 μm; b2, b4 100 μm; d1 to d4 200μm; inserts 25 μm.

PS1xAPP mice accumulated phospho-tau, LC3-II and ubiquitinated proteins in axonal dystrophies surrounding Abeta plaques (Figure 3; see also [15,16]). Thus, in agreement with the preservation of SOM/NPY neurons, lithium also reduced both the total AT8-positive phospho-tau levels, determined by western blots (Figure 3A), and the AT8-positive dystrophies, detected by immunohistochemistry (Figure 3B). Furthermore, lithium also reduced the steady-state levels of both LC3-II and ubiquitinated proteins (see Figure 3C). In fact, the steady-state levels of both LC3-II and ubiquitinated proteins were similar to those observed in the PS1 lithium group. Immunohistochemistry also demonstrated a dramatic reduction in both ubiquitin- and LC3-positive dystrophies, surrounding the Abeta plaques (Figure 3D, d2 and d4). Thus, lithium reduced the number of dystrophies and the accumulation of intracellular proteins.

This lithium-mediated reduction of the axonal dystrophic pathology could also ameliorate the synaptic degeneration. Thus, we determined (by western blots) the levels of the classic presynaptic marker synaptophysin (not shown). Control PS1xAPP mice displayed a consistent and significant reduction (0.72 ± 0.12 vs 1.00 ± 0.14 for PS1xAPP and PS1 control, respectively, n = 7 per phenotype, Tukey p < 0.05) whereas a completely recovery was detected in lithium-treated PS1/APP mice (0.96 ± 0.16 for PS1xAPP lithium, n = 9).

Taken together, these data indicated that the chronic lithium treatment markedly reduced the plaque associated axonal dystrophy pathology, in parallel with a reduction on the abnormal intracellular accumulation of proteins and/or autophagic vesicles, in this PS1xAPP model. Furthermore, lithium also decreased the putative presynaptic degeneration observed in this transgenic mouse model.

### Lithium treatment substantially modified the morphology and toxicity of the extracellular Abeta plaques

**Figure 4:**
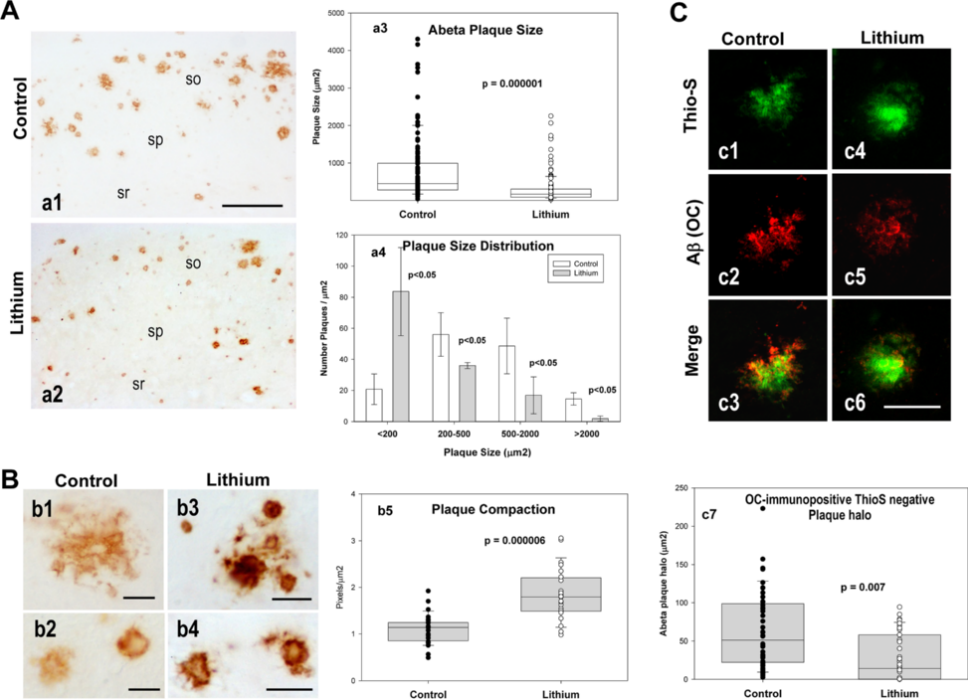
**Lithium treatment reduced the size and increased the compaction of the extracellular Abeta plaques. A)** Lithium treatment reduced the size of Abeta deposits. Plaques were immunostained with anti-Abeta42 antibody. Representative images (a1 and a2) and quantitative analysis (a3 and a4) of Abeta plaque size from the CA1 subfield of control and lithium-treated PS1xAPP mice. (a3) The individual plaque size (μm2) was quantitatively assessed from 25 sections of 6 different control or lithium treated PS1xAPP mice. (a4) The plaque size distribution was determined by calculating the number of plaques falling into distinct area categories (ranging from <200 μm2 to >2000 μm2). For each category, the difference between control and lithium was determined by t-test. so, stratum oriens; sp, stratum pyramidale; sr, stratum radiatum. **B)** Lithium treatment increased the Abeta plaque compaction. Signal density of Abeta42 immunostained plaques from control (b1, b2) and lithium-treated (b3, b4) mice were measured in the CA1 subfield of hippocampus. Optical densities (pixel/μm2) of plaques from 5 sections/mouse and 5 mice per group was represented in the graph. **C)** Plaque oligomeric halo, considered as the difference between OC- and Thio-S-stained areas was reduced by lithium. Plaques were sequentially staining with Thio-S and the OC antibody. (c1-c6) Representative images of a similar size plaque in the CA1 region of control (c1-c3) or lithium-treated (c4-c6) mice showing the plaque halo. (c7) Similar size plaques (< 200 μm2) were analyzed (15 plaques per animal, n = 3 mice per group). Scale bars: a1, a2 200 μm; b1-b4 25 μm; c1-c6 20 μm.

Next, we tested whether lithium treatment could alter Abeta accumulation in PS1xAPP hippocampus. Data (Table 1) indicated the absence of modifications on either i) total monomeric Abeta (quantified by western blot and 6E10 antibody), ii) soluble Abeta42 (quantified by ELISA using soluble extracts) or iii) Abeta plaque load (determined using either anti-Abeta42 or 6E10 antibodies). However, we did observe a highly significant reduction (−62.7%) in the size of the Abeta plaques in lithium-treated PS1xAPP mice, as compared with PS1xAPP control group (Figures 4A, a1, a2 and quantitatively in a3). In fact, when the distribution of the different sizes of the Abeta plaques was analyzed (Figure 4A, a4), the treated group displayed a marked increase (4 fold) in the number of small plaques (<200 μm2), with the consequent reduction in the number of medium and large size Abeta plaques.

**Table 1:** Abeta accumulation was not modified in the lithium treated PS1xAPP mice

		**Control**	**Lithium**
Soluble Abeta42	ELISA	27.57 ± 10.74	30.14 ± 6.47
(pg/ml)		n = 7	n = 7
Total Abeta	Western blots	1.0 ± 0.26	0.98 ± 0.60
(relative units)	6E10	n = 18	n = 25
Abeta load	Immunohistochemistry	6.83 ± 1.99	4.40 ± 1.81
(% of area)	6E10	n = 5	n = 5
Abeta42 load	Immunohistochemistry	5.09 ± 2.31	3.65 ± 1.03
(% of area)	Abeta42	n = 5	n = 5

Data are mean ± S.D.

This reduction of plaque size, in absence of a parallel reduction on the total Abeta deposition, could arise of a higher Abeta compaction. Interestingly, higher plaque compaction could reduce the Abeta pathology [33]. We thus determined the plaque compaction by quantifying the optical density of Abeta42 immunostained plaques, randomly selected by the stereological microscope (n = 800 plaques from 5 different sections and 6 different control or treated mice; see [33]). Although the plaque compaction was heterogeneous in both groups (Figure 4B), our data demonstrated the existence of a highly increase (82%) in the plaque compaction (calculated as Pixels/μm2) in the lithium treated PS1xAPP mice (Figure 4B, b5).

The increase in plaque compaction could involve a reduction of the putative toxic oligomeric Abeta that surrounded or aroused from plaques (the plaque “halo”; [34]. To quantitatively determine this possibility, the Abeta plaques were first stained with Thioflavin-S followed by immunostaining with the conformation-specific OC antibody, which recognizes fibrillar Abeta oligomers. Representative double labeled images, and the quantification of the plaque oligomeric halo, is shown in Figure 4C (c1 to c6, and c7, respectively). As expected, lithium treatment produced a significant reduction of the OC-positive plaque halo which might result in less toxic plaques.

**Figure 5:**
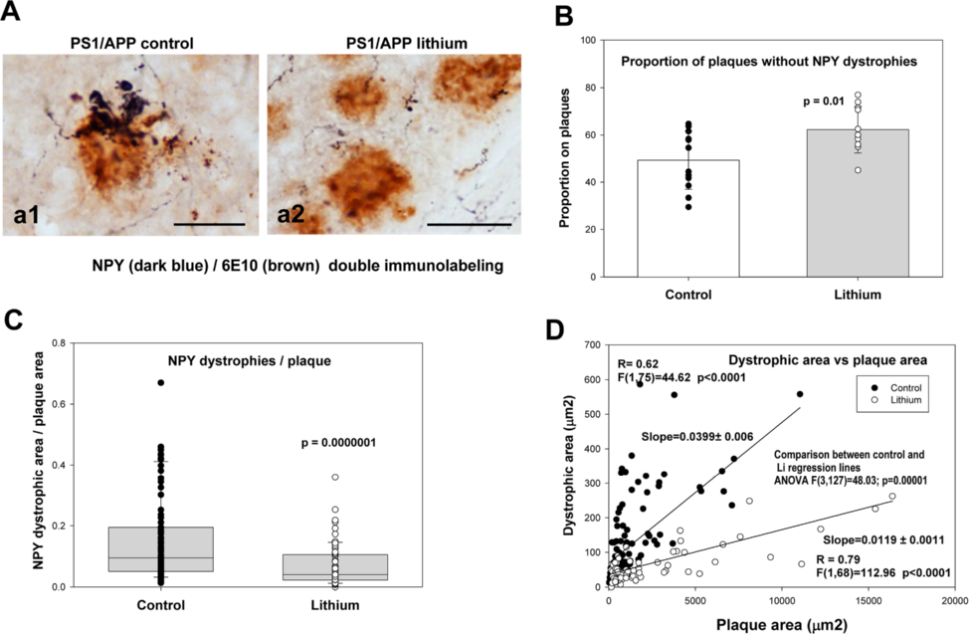
**Lithium treatment decreased the Abeta toxicity. A)** NPY-labeled dystrophic neurites, surrounding 6E10-labeled Abeta plaques, were compared in CA1 field of hippocampus from control (a1) and lithium-treated (a2) PS1xAPP mice (5 sections per mice, 3 mice per group). **B)** Lithium treatment increased the proportion of Abeta plaques displaying no apparent NPY-dystrophies. The difference between groups (indicated in the figure) was assessed using t-test. **C)** Lithium also significantly (using non-parametric Wilcoxon test) reduced the NPY dystrophic area, normalized by the corresponding plaque area. **D)** The decrease on Abeta toxicity, at all plaque sizes, was also assessed by plotting the individual NPY dystrophic area versus Abeta plaque area. The individual data from both groups were fitted to a linear model and the possible difference between groups was analyzed using Multiple Regression Analysis. As shown, both groups (control and lithium) could be fitted to a linear model and both regression lines were statistically different (ANOVA F(3,145) = 38.00; p = 0.00001). Furthermore, the slopes of the fitted data were also significantly different between both groups (Conditional Sum of Squares, see Results). Scale bars: a1 25 μm, a2 50 μm.

To examine the impact of lithium treatment on the toxicity of the Abeta plaques, we quantified the NPY dystrophic area and the corresponding Abeta area in individual plaques. Using unbiased stereological counts in NPY and 6E10 double immunostained sections (Figure 5A), we first calculated the proportion of Abeta plaques devoid of NPY dystrophies. As shown, treated PS1xAPP mice displayed a small, but significant, increase in the proportion of plaques without associated NPY dystrophies (Figure 5B). More relevant, when the NPY dystrophic area was normalized by the corresponding Abeta plaque area, we observed a substantial reduction of the NPY dystrophic area per plaque in the treated PS1xAPP mice (Figure 5C). Since this reduction of the dystrophic area could just reflect the decrease in plaque size, we also plotted single NPY-dystrophic area versus the corresponding Abeta plaque area, for both control and treated PS1xAPP mice. As expected, we observed a significant positive linear correlation between both parameters in both mice groups (Figure 5D). Importantly, we also observed a significant higher dystrophic area in control PS1xAPP mice across all size of plaques, as compared with treated PS1xAPP mice. In fact, the slope of the fitted linear regression, between dystrophic area versus plaque area, presented a 3-fold decrease after lithium treatment (0.0399 ± 0.006 vs 0.0119 ± 0.0011; for control and lithium PS1xAPP mice; ANOVA F(1,127) = 27.88, p = 0.00001).

Taken together, these data demonstrated that lithium treatment modified Abeta plaques quality decreasing their toxicity measured as the capacity to induce axonal dystrophies formation.

### Lithium treatment induced astrocyte activation and the incorporation of Hsps to Abeta plaques

**Figure 6:**
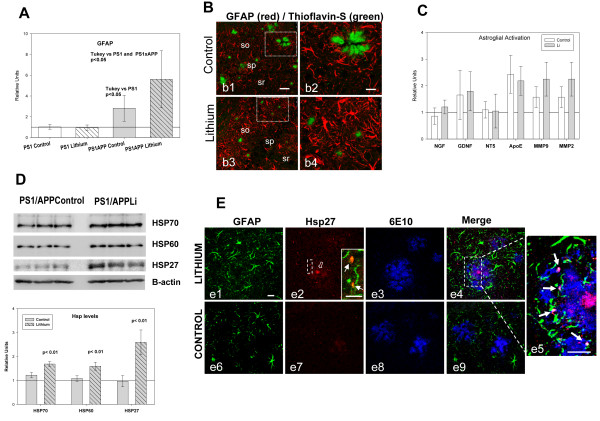
**Lithium increased the astrocyte activation and the Hsps levels. A-B)** Lithium treatment increased the astrocyte activation assessed by qPCR **(A)** and GFAP immunoreactivity **(B)**. GFAP expression **(A)** was assayed in hippocampal samples from 10 mice per group. GFAP expression increased in PS1xAPP control group and further increased in PS1xAPP lithium-treated mice. ANOVA (F(3,36) = 18.82, p = 0.0001). The post-hoc analysis using Tukey is presented in the graph. **B)** Representative confocal images through the CA1 field of control (b1, b2) and lithium-treated (b3, b4) mice, showing GFAP-immunoreactive astrocytes (red) and Thioflavin-stained Abeta plaques (green). As shown, GFAP immunoreactivity is clearly higher in lithium-treated mice. **C)** Lithium did not modify the expression of NGF,GDNF, NT-5, MMP9, MMP3 or ApoE, determined by qPCR (n = 4 mice per group). **D)** Lithium treatment increased the levels of three Hsps (Hsp70, Hsp60 and Hsp27). Representative western blots and quantitative analysis of Hsps using protein extract from hippocampus of control and lithium-treated PS1xAPP mice (n = 4 mice per group). The expression of all three Hsps was normalized using PS1 control (not shown), and the significance was determined by t-test. **E)** Confocal images through the CA1 subfield showing GFAP/Hsp27/Abeta triple immunofluorescence labeling. In lithium-treated mice (e1-e5), the core of the Abeta plaques (labeled by 6E10, in blue) is intensely stained with the anti-Hsp27 antibody (in red), as well as astrocytic puncta processes (labeled by GFAP, in green) surrounding the plaques (see insert in e2 showing double GFAP-Hsp27 labeling, and the triple labeling detail in e5). On the contrary, in control mice (e6-e9), the core of the Abeta plaques appears almost devoid of Hsp27 immunostaining and low or no immunoreactivity was observed in astrocytes surrounding the plaques. Scale bars, d1-d9 20 μm, insert in a2 10 μm.

Finally, we investigated the possible implication of astrocytes on this lithium-mediated modification of Abeta plaques. Activated astrocytes, surrounding Abeta, could highly influence the Abeta compation and plaque aggregation [35-37]. Thus, we evaluated whether lithium affected the astrocyte activation by determining the expression of GFAP by qPCR (Figure 6A) and immunohistochemistry (Figure 6B, b1 to b4). As expected, GFAP expression and astrocyte activation were increased in control PS1xAPP group (as compared with PS1 mice, Figure 6A and B). As also shown, lithium treatment produced a higher increased in GFAP expression (Figure 6A) and also in GFAP activation (compare Figures 6B, b2 and b4). In fact, in the treated PS1xAPP mice, astrocytes were more immunoreactive and clearly hypertrophic, as compared with control PS1xAPP. However, no significant lithium-dependent differences were detected on the expression of other factors, such as NGF, GDNF, NT-5, MMP9, MMP3 or ApoE, (Figure 6C). It is also noteworthy that lithium treatment has no effect on GFAP expression on non-Abeta activated astrocytes (PS1 lithium mice).

Within the different factors and/or processes that could influence Abeta compaction, it has been demonstrated that extracellular chaperones, such as heat shock proteins (Hsps), have the capacity to reduce the Abeta toxicity by increasing the sequestration/compaction of putative toxic Abeta oligomers [36,38-41],. Furthermore, it has been also demonstrated that astrocytes could express and release different Hsps [36]. Thus, we evaluated whether lithium treatment did affect the Hsps expression. First, we determined, by western blots, the levels of Hsp27, Hsp60 and Hsp70 in PS1xAPP control and lithium treated mice. As shown in Figure 6D, we observed a consistent increase on the levels of all four proteins in the lithium group. However, no changes on expression (by qPCR) were detected (not shown). Moreover, we analyzed the in vivo localization of Hsp70 (not shown) and Hsp27 (Figure 6E) and whether lithium treatment modified their distribution. Both chaperones displayed similar immunostaining patterns. In lithium treated PS1xAPP mice (Figure 6E e1 to e5), triple labeling experiments demonstrated that anti-Hsp27 intense stained the Abeta plaque core (labeled by 6E10) and, interestingly, also activated astrocytes (GFAP-positive cells) near plaques displayed Hsp-27 immunopositive puncta. However, in control PS1xAPP mice, the core of Abeta plaques appeared weakly immunostained and low or no immunoreactivity was observed in astrocytes surrounding Abeta plaques (Figure 6E, e6-e9).

Although more experiments are clearly needed, these data indicated that lithium, modulating the production/release of Hsps by astrocytes, might decrease the toxicity of plaques by increasing the Abeta compaction.

## Discussion

Here, we demonstrate that chronic oral administration of lithium, before the pathology onset, resulted in less toxic plaque formation that significantly ameliorated the degenerative processes and behavioral/memory deficits occurring during disease progression in our PS1xAPP model. Specifically, and of great relevance for AD prevention, early lithium intervention was able to arrest neuronal loss in hippocampus and entorhinal cortex of highly vulnerable populations, Beside, lithium substantially reduced the axonal dystrophic pathology, associated to amyloid plaques, by increasing the Abeta compaction. As we discuss below, these neuroprotective effects of lithium could be mediated by modifications of the plaque toxicity through the astrocytic release of heat shock proteins. On contrary to previous failed clinical studies using lithium, our results highlight the potential use of this compound as a preventive intervention to halt/slow AD pathology progression at preclinical stages.

As we reported previously, our PS1xAPP mouse displays early (6 months) neuronal loss affecting SOM/NPY GABAergic cells in the hippocampal formation and entorhinal cortex, which coincides spatiotemporally with the extracellular Abeta deposition [15,16,23,24,30,31]. In addition, another pathological feature of this AD model is the formation of abundant axonal dystrophies surrounding the Abeta plaques. Dystrophies accumulated phosphorylated tau, ubiquitinated proteins and autophagic vesicles [15,16].

Our data demonstrate that lithium administration, starting before the beginning of the neurodegenerative processes, avoids the selective neuronal loss of the SOM/NPY cells in both the hippocampus and entorhinal cortex. This is the first report showing that lithium prevents neuronal loss in AD vulnerable brain regions using in vivo studies. Also, our data demonstrate that lithium ameliorates the dystrophic pathology, reducing dramatically the NPY-positive dystrophic area associated to the Abeta plaques and decreasing the levels of abnormally accumulated LC3-II, AT8 and ubiquitinated proteins. Thus, lithium clearly alleviates most of the neuropathological signs of the PS1xAPP model.

As we and others have demonstrated, the PS1xAPP mice display GSK-3beta activation and autophagy/lysosomal deficiencies [15,16,31,42,43]. Lithium could directly affect the neuronal degeneration by inhibiting the GSK-3beta activity (data not shown; [44]) and/or by activating the autophagy-mediated protein degradation [12,45,46]. These effects would reduce the accumulation of phospho-tau, LC3-II and ubiquitinated proteins and, in consequence, reduce the neurodegenerative process. However, the PS1xAPP transgenic model accumulates these proteins in axonal dystrophies surrounding the Abeta plaques. In this sense, we and others have suggested that the formation of axonal dystrophies might be directly implicated in the neuronal degeneration during disease progression [9,15,16,47,48]. This suggestion agrees with recent data from AD patients [49]. Importantly, quantitative data demonstrate that lithium produced a prominent reduction (−60%) of the NPY-positive dystrophic area. This reduction could also be reflected by the decrease in the abnormal accumulation of phospho-tau, LC3-II and ubiquitinated proteins, associated with the dystrophic pathology, surrounding Abeta plaques.

Therefore, besides a putative direct effect either through GSK-3beta activity or autophagy/lysosomal protein degradation, the lithium-mediated amelioration of the neuropathological alterations may likely reflect the dramatic reduction in the formation of dystrophic neurites around the Abeta plaques. This effect could also reflect the lithium-dependent modifications of the Abeta plaque formation.

As we have shown here, lithium produced a prominent change in plaque morphology and quality. In fact, the Abeta plaques were smaller (see also [20]) and more compact in treated than in control PS1xAPP mice. In this context, it has been reported that the highly aggregated Abeta possesses a reduced toxicity [33,50], and therefore the observed decrease in the dystrophic area per plaque could reflect a reduction in plaque toxicity.

Regarding the plaque toxicity, it has been noted that the formation of axonal dystrophies and the synaptic degeneration seemed to be restricted to the periphery of the Abeta plaques [34,48,50]. This most periphery area (halo) of the plaques might be constituted by partially aggregated Abeta fibrillar oligomers, which could be involved on the AD pathology [51]. In this scenario, our data demonstrate that lithium produces a reduction on the fibrillar oligomeric halo (which is recognized by the conformation specific polyclonal OC antibody) of the Abeta plaques, thus diminishing the plaque toxicity.

The processes contributing in the Abeta aggregation, plaque formation or plaque compaction are actually unknown. It has been suggested that astrocytes could play a prominent role by limiting the plaque growth and the plaque-associated dystrophy formation [35,36]. Moreover, activated astrocytes may release, among different factors, Hsps [52,53], which could induce the Abeta aggregation, reducing its potential toxicity [36,38,40]. In agreement with these data, our results strongly suggest the involvement of astrocytes and extracellular Hsps as mediators of the lithium effect on plaque toxicity. In fact, we demonstrate simultaneous higher astrocyte activation with higher incorporation of Hsps in the Abeta plaques and reduced oligomeric plaque halo in lithium treated PS1xAPP mice, compared with controls. It is noteworthy the lithium-dependent increase of Hsp70 and Hsp27 in the plaque core. Although further experiments should be done, this particular localization suggests that these Hsps could be implicated in the Abeta nucleation and plaque compaction.

## Conclusions

Our data demonstrate that the early chronic lithium treatment significantly ameliorates the pathological progression in this PS1xAPP AD model. Lithium could reduce neuronal/axonal degeneration by increasing the Abeta compaction and, in consequence, producing smaller Abeta plaques with lower toxic halo. Lithium could influence directly neurons but, as we have shown in this work, this compound has a novel therapeutic effect through astrocytes inducing chaperones release which have the capacity to modulate the Abeta compaction/toxicity. To the best of our knowledge, this is the first time that this therapeutic effect of lithium on Abeta plaque quality has been reported. These data reveal a novel lithium-mediated mechanism capable of altering the course of the disease in an amyloidogenic AD model. These Abeta-modifying mechanism might represent an innovative therapeutic approach to the, so far, continuing negative outcomes of AD clinical trials aimed to clear Abeta plaques once they have already formed, and to the current inability to prevent plaques from forming in the first place.

## Competing of interests

The authors declare that they have no competing of interests.

## Authors’ contributions

MT, SJ, VN and MV carried out the molecular experiments; R S-V, L T-E, and E M-S carried out the immunohistochemical experiments; D B-V and I M-G carried the stereological experiments; V DC performed the behavioral analysis; JCD and MV participated in the design of experiment and revising the manuscript, AG and JV design the experiments, analyzed the data and wrote the manuscript. All authors read and approved the final manuscript.
